# Hidden Genetic Regulation of Human Complex Traits via Brain Isoforms

**DOI:** 10.1007/s43657-023-00100-6

**Published:** 2023-03-20

**Authors:** Lu Pan, Chenqing Zheng, Zhijian Yang, Yudi Pawitan, Trung Nghia Vu, Xia Shen

**Affiliations:** 1 Biostatistics Group, School of Life Sciences, Sun Yat-Sen University, Guangzhou, 510006 China; 2 Department of Medical Epidemiology and Biostatistics, Karolinska Institutet, Stockholm, 17177 Sweden; 3 State Key Laboratory of Genetic Engineering, School of Life Sciences, Fudan University, Shanghai, 200433 China; 4 Center for Intelligent Medicine Research, Greater Bay Area Institute of Precision Medicine (Guangzhou), Fudan University, Guangzhou, 511458 China; 5 Centre for Global Health Research, Usher Institute, University of Edinburgh, Edinburgh, EH8 9AG UK

**Keywords:** Alternative splicing, Isoform-ratio quantitative trait loci (irQTL), Expression quantitative trait loci (eQTL), Genome-wide Association Studies, Neuro-related human complex traits

## Abstract

Alternative splicing exists in most multi-exonic genes, and exploring these complex alternative splicing events and their resultant isoform expressions is essential. However, it has become conventional that RNA sequencing results have often been summarized into gene-level expression counts mainly due to the multiple ambiguous mapping of reads at highly similar regions. Transcript-level quantification and interpretation are often overlooked, and biological interpretations are often deduced based on combined transcript information at the gene level. Here, for the most variable tissue of alternative splicing, the brain, we estimate isoform expressions in 1,191 samples collected by the Genotype-Tissue Expression (GTEx) Consortium using a powerful method that we previously developed. We perform genome-wide association scans on the isoform ratios per gene and identify isoform-ratio quantitative trait loci (irQTL), which could not be detected by studying gene-level expressions alone. By analyzing the genetic architecture of the irQTL, we show that isoform ratios regulate educational attainment via multiple tissues including the frontal cortex (BA9), cortex, cervical spinal cord, and hippocampus. These tissues are also associated with different neuro-related traits, including Alzheimer’s or dementia, mood swings, sleep duration, alcohol intake, intelligence, anxiety or depression, etc. Mendelian randomization (MR) analysis revealed 1,139 pairs of isoforms and neuro-related traits with plausible causal relationships, showing much stronger causal effects than on general diseases measured in the UK Biobank (UKB). Our results highlight essential transcript-level biomarkers in the human brain for neuro-related complex traits and diseases, which could be missed by merely investigating overall gene expressions.

**Supplementary Information:**

The online version contains supplementary material available at 10.1007/s43657-023-00100-6.

## Introduction

Alternative splicing is an essential mechanism in diversifying the genetic and proteomic landscapes in eukaryotes (Thakur et al. 2019). It is a crucial process by which proteins with different functions are produced from a single gene through patterned joining and excising of the introns and exons within the gene (Nature Portfolio 2022). Alternative splicing occurs in around 95% of multi-exonic genes in humans (Nilsen and Graveley 2010; Pan et al. 2008), and is subjected to tissue-specific regulations (Zaghlool et al. 2014). It is a process that is most complex in the nervous system (Yeo et al. 2004). In the central nervous system, our brain has the highest level of alternative splicing out of all tissues in the human body (Porter et al. 2018; Xu et al. 2002).

Genetic regions that control and regulate these alternative splicing events, often known as splicing quantitative trait loci (sQTL), have been successively discovered in large-scale genome-wide association studies (GWAS) (Ardlie et al. 2015; Battle et al. 2014; Park et al. 2018), and recent studies have shown sQTL landscapes in human brain sub-tissues (Takata et al. 2017; Walker et al. 2019; Zhang et al. 2020). These studies used percent spliced index (PSI, ratios between exon-included and exon-excluded reads) values (Schafer et al. 2015) calculated directly from RNA-sequencing data to obtain splicing scores representing splicing patterns in genes prior to sQTL discoveries. Expressed isoform ratios per gene were also considered as phenotypes in mapping sQTL (Lappalainen et al. 2013). However, directly mapping isoform level quantitative trait loci (QTL) has always been challenging due to the difficulty in quantifying isoform expressions using short RNA-sequencing reads.

Here, we aim to discover the QTL regulating expressed isoform ratios per gene (i.e., irQTL) across brain tissues, where the isoform expressions were quantified using our previously developed isoform expression estimation method, X-matrix alternating expectation–maximization (XAEM) (Deng et al. 2019), which outperforms the other state-of-the-art methods in isoform estimation. We quantify isoform ratios in multi-isoform genes in 1,191 samples from 13 brain tissues and carry out GWAS analysis of the isoform ratios with the genotyping data to acquire cis-irQTL. These irQTL regulate the relative proportions across the isoforms per gene instead of the overall gene expression. We show that genes with such irQTL genetic basis in the brain contribute significantly to neuro-related phenotypes.

## Materials and Methods

### Samples Origin and Data Acquired

#### RNA Sequencing and Whole-Genome Sequencing Data

RNA sequencing data used in this study were obtained from the genotype-tissue expression (GTEx) project (Ardlie et al. 2015) portal (https://www.gtexportal.org, version phs000424.v7.p2.c1). We considered 13 brain tissues, consisting of 1,236 RNA sequencing samples from 172 individuals from the GTEx project (Fig. 2a). As an individual might die from different causes, the tissue(s) sampled from the individual was from diseased-free sampling sites. GTEx donors are aged between 21 and 70 with the following criteria exclusion criteria: individuals with human immunodeficiency virus (HIV) infection or high-risk behaviors, viral hepatitis, metastatic cancer, chemotherapy or radiation therapy for any condition within the past two years, and whole-blood transfusion in the past 48 h or body mass index $>35$ or $<18.5$ (Ardlie et al. 2015).

Whole-genome sequencing (WGS) data for these individuals were obtained from the GTEx portal under version phs000424.v7.p1. There are 6,496,708 markers called from WGS, including 5,987,177 SNPs and 509,531 InDels. The final sample size used for QTL analysis is 1,191 samples, for which both RNA-sequencing and whole-genome sequencing data are available.

#### GTEx cis-eQTL Data

Cis-eQTL are genomic loci near the corresponding coding genes that explain a fraction of the genetic variance of the gene expression phenotypes (Glass et al. 2013). GTEx has summarized a list of cis-eQTL for each tissue type in its data portal. In this study, only cis-eQTL from the brain tissues were considered.

### RNA-Sequencing Mapping and Quantification

We acquired demultiplexed raw RNA sequencing FASTQ files from the GTEx portal and used the fast mapping and isoform quantification tool XAEM (Deng et al. 2019), which has higher accuracy than popular methods such as Salmon (Patro et al. 2017) and Kallisto (Bray et al. 2016), to process the data for RNA sequencing alignment and isoform expression quantification. Mapping was performed using XAEM V0.1.0 with reference to the human reference genome hg19/GRCh37 (UCSC hg19 annotation). Isoform quantification was done subsequently using the default setting in XAEM to produce isoform counts, normalized to transcripts per million (TPM) values, for each sub-brain tissue. Mapping and quantification were carried out separately for each brain tissue.

### Quality Control Measures

Quality control was carried out for isoform expression data after quantification using XAEM software. We considered in total 24,629 genes with 46,710 isoforms based on the human reference genome hg19/GRCh37. To identify the genetic regulation of isoform expression missed by eQTL analysis, we considered only multi-isoform genes, which led to a total of 9,401 genes with 31,482 isoforms after filtering. Of these 9,401 genes, 8,382 of them are protein-coding genes, and the rest includes lncRNA, antisense-RNA, pseudo-genes, etc. (Supplementary Table 3). Individuals with half or more than half of their genes having zero counts were removed for subsequent analyses, which reduced the original 1,671 viable samples in GTEx to the 1,236 retained samples we started with. For the principal component analysis (PCA) and QTL mapping, only variants with minor allele frequency (MAF) $>0.05$ were considered, which resulted in 6,164,423–6,316,616 variants across the brain tissues.

### irQTL Mapping

In this study, we identify irQTL for 13 brain tissues. For genes with multiple isoforms, isoform ratios were defined as TPM values of isoforms divided by their respective gene-level TPM. PCA was carried out on the genomic kinship matrix constructed via the whole-genome sequencing genotype data using PLINK (Purcell et al. 2007), to obtain a set of genomic principal components (PCs) to be used as covariates in the subsequent association scan. Age, sex, and the first three PCs were then used as covariates, whose effects were taken away from the isoform ratios using linear regression. The resulting values were used as covariates-corrected isoform ratios for downstream analyses. We performed cis-regulatory region association analysis by regressing the isoform ratio phenotypes on the genotype data using RegScan V0.5, a GWAS analysis tool for linear regression analysis with continuous traits maximally fast on large data sets with many phenotypes (Planell et al. 2021).

### Locus Definition

Each cis-regulatory locus, as well as the irQTL region, was defined as the $\pm$ 1 Mb region around the corresponding gene. The SNP with the lowest *p*-value within each locus was selected as the lead variant, and the associations having $p<5\times {10}^{-8}$ were retained for the subsequent analyses. These significant irQTL from each brain tissue were then compared with the cis-eQTL by the GTEx Consortium of the corresponding gene. We focused on the irQTL with eQTL *p*-values greater than 0.05 as the final set of irQTL.

### Stratified Linkage Disequilibrium Score Regression (S-LDSC)

We used S-LDSC (Bulik-Sullivan et al. 2015) to test whether the annotated genic regions are enriched for heritability of a certain trait, where the GWAS summary statistics were available via linkage disequilibrium (LD) hub (LD-Hub) (Zheng et al. 2016). The summary statistics were harmonized by the munge_sumstats.py procedure of the LDSC software. LD scores of HapMap3 SNPs (Altshuler et al. 2010) (major histocompatibility complex region excluded) for the annotated genes in each brain tissue were pre-computed using a 1-cM window (default). The heritability enrichment in each tissue was evaluated by an enrichment score, defined as the proportion of heritability captured divided by the proportion of annotated SNPs. The LDSC-v1.2 baseline annotations were fitted as covariates as LDSC suggested (Bulik-Sullivan et al. 2015; Gazal et al. 2017), which controls the residual variance in the chi-squared statistics and thus produces a more robust estimation of heritability enrichment on our desired annotation. For each tissue, we ran a separate model to test the heritability enrichment at the tissue-specific irQTL. This fits our hypothesis testing purpose, meanwhile avoiding potential multi-collinearity due to the similarity across brain tissues.

### Mendelian Randomization (MR) Analysis

Prior to the analysis, we extracted 152 neuro-related traits from LD-Hub and 200 UK Biobank (UKB) diseases with international classification of diseases (ICD) codings from Neale’s lab GWAS results. From the original 152 neuro-related traits, we removed the duplicated traits from different sources and highly correlated traits e.g., defined by alternative phenotype codings. This resulted in 114 neuro-related phenotypes for subsequent analysis. We conducted an MR analysis between the isoform ratios and 114 neuro-related traits and the 200 UKB diseases using the standard inverse-variance weighted (IVW) method for all the cis-irQTL and the MR Egger regression (Bowden et al. 2017) for the cis-irQTL with at least three independent instruments after LD-clumping (${r}^{2}<0.001$). Here, the cis-irQTL were used as genetic instruments, and the coding alleles were matched between the exposure isoforms and the outcome phenotypes before estimating potential causal effects.

## Results

**Fig. 1:**
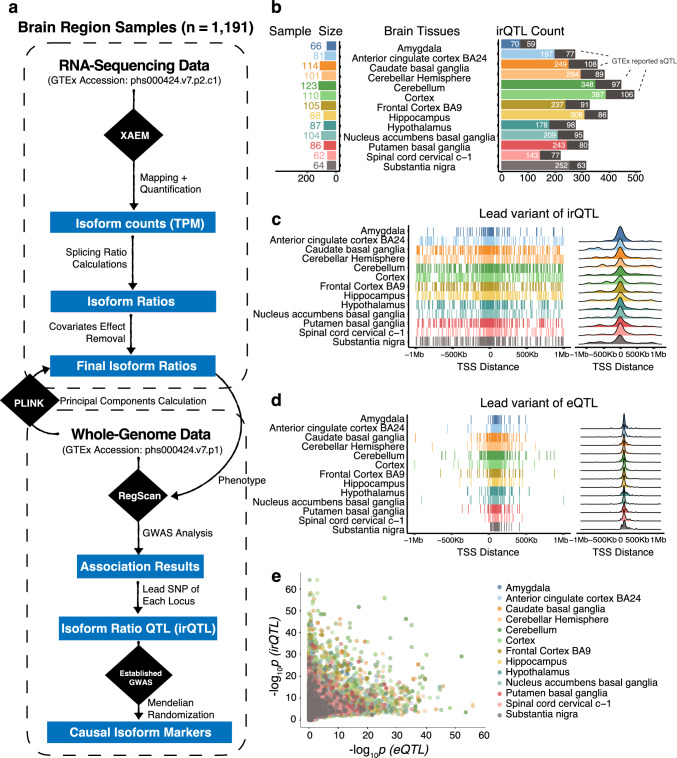
irQTL workflow and summary statistics. **a** RNA sequencing data of 1,191 samples from 13 brain regions were obtained from the GTEx Consortium. Alignment and isoform quantification were analyzed using the XAEM software based on TPM for each sample. For multi-isoform genes, the isoform ratio for each isoform was calculated as the TPM value for each isoform divided by the overall corresponding gene expression TPM value. PLINK was used for calculating the genomic kinship matrix and three PCs. Fixed effects of age, sex, and the first three PCs were removed from the isoform ratios phenotypes. The phenotypes were inverse-Gaussian transformed prior to GWAS analysis using RegScan. The lead variants of the mapped irQTL were passed onto subsequent analysis, including causal inference referencing the PhenoScanncer database. **b** The sample size in irQTL mapping and the corresponding detected irQTL count for each brain tissue, where the number that overlaps with GTEx-reported sQTL ($p<5\times {10}^{-8}$ is marked. **c** Lead variants locations of the mapped irQTL ($p<5\times {10}^{-8}$) with respect to their distance to the transcription start sites (TSS). **d** Lead variants locations of the mapped eQTL ($p<5\times {10}^{-8}$) with respect to their distance to the transcription start sites (TSS). **e** $-{\mathrm{log}}_{10}p$ values comparison between irQTL and eQTL, where the 4,241 irQTL signals (${p}_{irQTL}<5\times {10}^{-8}$, and ${p}_{eQTL}>0.05$) were annotated to test for heritability enrichment

We aim to identify irQTL in 13 sub-brain tissues, where RNA and DNA sequencing data are both available in 1,191 GTEx consortium samples. The overall workflow is illustrated in Fig. 1a. After RNA sequencing reads mapping and quantification, isoform counts were estimated using XAEM for each sub-brain tissue. For the multi-isoform genes, the isoform ratios were defined as the isoform TPM values divided by their corresponding gene-level TPM values. Cis-regulatory region association analysis was performed on each isoform ratio phenotype, where fixed effects including sex, age, and the first three genomic principal components (PCs) were corrected for the phenotype. The corrected phenotypic values were also used in downstream analyses.

The association analysis identified 7,099 cis-irQTL in the brain ($p<5\times {10}^{-8}$, equivalent to an estimated false discovery rate (FDR) of $9.6\times {10}^{-5}$ to $5.1\times {10}^{-4}$ across different tissues), where 4,241 of those did not show any effect as gene expression quantitative trait loci (eQTL) on general gene expression levels (${p}_{eQTL}>0.05$) (Fig. 1b,e). For these irQTL, the corresponding genes have relatively stable expression levels in the brain tissues, but the proportions of their isoforms are genetically regulated by the irQTL. Taking the frontal cortex as an example, 493 irQTL were detected for 265 genes whose overall expressions do not show eQTL effects (Fig. 1b, Supplementary Table 1). We cross-referenced these detected irQTL in the latest sQTL results using sQTLseekeR (Monlong et al. 2014) by the GTEx Consortium. Based on the same significance threshold ($p<5\times {10}^{-8}$), GTEx reported 1,126 irQTL out of the total 4,241 irQTL as sQTL (Fig. 1b, Supplementary Table 3). We also cross-referenced the detected irQTL in the sQTL reported by the recent testing for heterogeneity between isoform-eQTL effects (THISTLE) method. As THISTLE utilizes a heterogeneity test per gene for sQTL discovery, we compared the sGene (genes with significant splicing QTL) counts between the irQTL and THISTLE sQTL results. Overall, the discovered irQTL mapped to 874 sGenes, where 96 overlap with THISTLE sGenes.

The majority of the detected irQTL lead variants are centered at the transcription start sites (TSS) (Fig. 1c), which is a feature also seen in eQTL (Fig. 1d) and even protein QTL (pQTL) in the human plasma (Sun et al. 2018). Nevertheless, we found that the lead variants of cis-eQTL were generally more condensed around the TSS of the corresponding genes than those of cis-irQTL. This could be caused by two reasons: (1) different isoforms of the same gene had different regulatory elements for their transcription; (2) the isoform expressions were estimated and thus had lower statistical power compared to the corresponding overall gene expressions given the same sample size, as unlike for gene expression where one could count the sequencing reads for quantification, the shared reads between isoforms provide incomplete information for isoform expression.

**Fig. 2:**
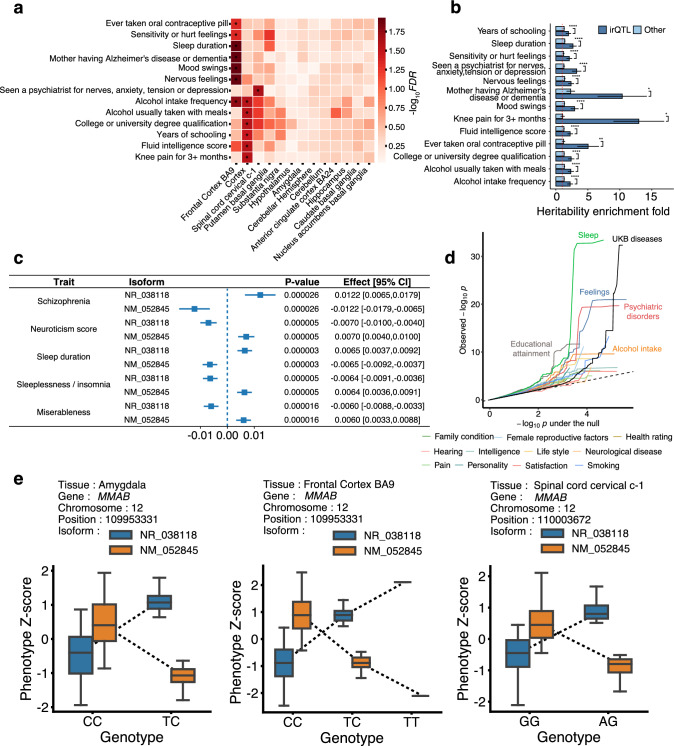
Genetic effects on complex traits mediated through irQTL.** a** Complex traits heritability enrichment signals at the irQTL regions, where the significant enrichment (FDR < 5%) results were highlighted. **b** Comparison of the heritability enrichment at irQTL genes and other genes for the significant traits in (**a**). **c** Example of the MR results for gene *MMAB*, whose two isoforms have genetically regulated ratios in different tissues, showing plausible causal effects on multiple neuro-related phenotypes. **d** Quantile–quantile plot for the significance of all the MR causal effects tests in 114 neuro-related traits (in categories) and 200 UKB diseases. **e** Genotype–phenotype map of the significant irQTL of *MMAB*, as instruments for the MR discoveries for neuro-related phenotypes in (**c**). In each case, the normalized isoform ratios of the two isoforms of the same gene sum up to similar values across different genotypes; thus the irQTL could not be mapped as significant eQTL

In each brain tissue, we annotated the genomic regions for the corresponding irQTL and applied S-LDSC (Bulik-Sullivan et al. 2015) to estimate and test for heritability enrichment of complex traits. We considered the 114 neuro-related traits (Supplementary Table 4) whose GWAS summary statistics are available through LD-Hub (Zheng et al. 2016). Across the 13 (tissues) $\times$ 114 (traits) = 1,482 enrichment tests, the distribution of the S-LDSC reported *p*-values significantly deviated from the null (Supplementary Fig. 5). With FDR of less than 0.05, three brain tissues were found to be significantly associated with 13 neuro-related traits via the genetic regulation of isoform proportions per gene instead of gene expression levels (Fig. 2a), as the genome annotation of the detected irQTL does not carry any nominal eQTL effect. Such heritability enrichment on irQTL genes was also significantly higher than that on the other coding genes (Fig. 2b). The frontal cortex (BA9) was associated with Alzheimer’s or dementia, mood swings, nervous feelings, sensitivity or hurt feelings, sleep duration, alcohol intake, and contraceptive pill intake; the cortex was found to be associated with educational attainment, alcohol intake, intelligence, and knee pain; the cervical spinal cord was found to be connected to anxiety or depression.

We subsequently extracted the established genotype–phenotype association records of the corresponding irQTL LD-clumped (${r}^{2}<0.001$) significant variants from the same set of 114 neuro-related traits. We conducted inverse-variance weighted (IVW) MR analysis for all the isoform-trait pairs and an MR Egger regression (Bowden et al. 2017) for the irQTL with at least three instrumental variants after LD-clumping (Supplementary Table 5). This procedure revealed 1,092 isoform-trait pairs with plausible causal relationships (FDR less than 0.05, Supplementary Table 5). For comparison, we also conducted the same analysis for 200 UKB diseases with ICD codes whose GWAS summary statistics were publicly available from Neale’s lab. This procedure revealed 250 isoform-disease pairs with plausible causal relationships (FDR less than 0.05, Supplementary Fig. 3, Supplementary Table 6). As most of the 200 UKB diseases are not neuro-related, we found, as expected, generally stronger signals of MR discoveries for the neuro-related traits than for the UKB diseases; for instance, the causal inference discoveries were enriched for the traits categories including educational attainment, sleep, psychiatric disorders, feelings, and alcohol intake (Fig. 2d). Taking the gene *MMAB* with the most MR discoveries as an example, its irQTL could be mapped in multiple tissues, leading to the downstream causal inference of its isoforms on multiple neuro-related phenotypes, such as sleep duration, insomnia, neuroticism, miserableness, and schizophrenia (results in the amygdala are illustrated in Fig. 2c). Reverse causal inference analysis did not reveal statistically significant effects of the complex traits on the isoform expressions (Supplementary Fig. 4). Vitamin B12 is involved in the production of sleep-regulating neurotransmitter melatonin (Hashimoto et al. 1996; Mayer et al. 1996), and the protein product of *MMAB* was reported to catalyze the conversion of vitamin B12 into its final product adenosylcobalamin (Safran et al. 2021). For the two isoforms of *MMAB* quantified via the XAEM algorithm, the genotype–phenotype maps of its irQTL in different tissues illustrated that these isoform-level mediators could not be detected in the standard eQTL analysis (Fig. 2e), as not the gene expression themselves but rather the relative proportions between the isoforms regulated the downstream phenotypes.

## Discussion

We have conducted a series of investigations for brain irQTL, i.e., the cis-regulatory loci in the brain tissues that control the relative isoform proportions per gene instead of the expression levels. Besides identifying hundreds of irQTL that could not be detected as eQTL, we found that genes with such irQTL regulatory property harbor enriched heritability for human complex traits, especially here for neuro- or nerve-related phenotypes. We also inferred that genetically regulated isoform distributions have downstream effects on the phenotypes via MR. Our analysis highlights the importance of quantifying and studying isoform expressions rather than general gene expressions. Some genetically regulated functional transcripts may only be detected when the isoforms are adequately quantified.

We used our previously developed XAEM algorithm to estimate isoform expression in the GTEx brain tissue samples. Although the isoform expression level could not be directly obtained from RNA sequencing reads, the XAEM algorithm allows powerful quantification of isoform expression for multi-isoform genes. The estimated isoform expressions allowed us to demonstrate the regulation of gene expressions that could not be well characterized without dissecting into isoforms. For isoform expression estimation, transcriptome annotation reference is a factor to be considered. The more comprehensive references, such as Ensembl (Cunningham et al. 2021) and GENCODE (Frankish et al. 2020), contain much more transcripts, and many of them are not curated isoforms. Many exons in these references for a gene are only a few bases different from others, making some isoforms very similar to each other. Too many of too similar isoforms in the cluster response profile (CRP; the X matrix) of XAEM would worsen the estimation. This is true for any isoform quantification algorithm that relies on an isoform annotation reference, as it is difficult to have sequencing reads that well distinguish very similar isoforms. For normal analysis such as this study, it is better to use curated isoforms in RefSeq (O'Leary et al. 2015) (default CRPs inbuilt in XAEM), so that the results are more reliable.

The mapped irQTL could not be identified as a typical eQTL since the genetic effects on different isoforms per gene had different signs. Thus, although the genetic effects on different isoform expressions for the same gene were all strong, the gene’s overall expression could still be consistent across individuals with different genotypes (lacking genetic variance). This phenomenon can be generalized to other composite phenotypes too. In general, we would need to go deeper into the specific genetically regulated phenotype (in this case, isoform expressions) instead of only studying the composite phenotype (in this case, overall gene expressions).

We decided only to use age, sex, and three PCs as covariates mainly due to two perspectives: (1) we aimed to control the number of covariates in such small-sample association analysis to save degrees of freedom, as long as the inflation factor can be well controlled, and we found that the current setting was sufficient (inflation factor $\lambda =1.030$ at the median and $\lambda =1.018$ at the 25% quantile, Supplementary Fig. 1); (2) although the RNA sequencing data here are from multiple tissues, they are all from the brain, and the technical and biological conditions are less heterogeneous comparing to experiments in other tissues used in GTEx. Considering these, we did not consider more PCs or other covariates. In general, in small-sample genetic association analysis, the trade-off between degrees of freedom and power is a concern.

It is essential to clarify the difference in mapping ordinary sQTL and irQTL. First, sQTL mapping tools such as sQTLseekeR (Monlong et al. 2014) (used by the GTEx sQTL analysis), LeaftCutter (Li et al. 2018) (annotation-free), and the recently developed THISTLE method (Qi et al. 2022) (annotation-based isoform-level genetic effects heterogeneity test) aim to detect genetic regulation of “alternative splicing events”, namely, whether alternative splicing happens more for a certain genotype. Studying the isoform expressions as phenotypes themselves was not straightforward, and the main reason behind this is the great challenge in estimating isoform expressions using short-read RNA-sequencing data. Our initial idea of this work is to emphasize that isoform expression itself can be treated as an analyzable phenotype, as long as the estimation accuracy is sufficient. XAEM is a tool that substantially improves isoform expression estimation and thus fits the purpose. Although the estimation is not perfect, we showed that a number of novel isoform-level expression QTL could be mapped, which were missed in eQTL mapping (which neglects alternative splicing) and standard GTEx sQTL analysis (which does not have comparable isoform expression estimation). We would like to note that the comparison between irQTL and sQTL is subject to current statistical power. A slight difference in the genetic effects of irQTL for the isoforms of the same gene would be detected as sQTL when the power grows; nevertheless, as long as the isoform expressions can be well quantified (e.g., more commonly in the future with long-read sequencing techniques), studying isoform expressions as phenotypes would directly give us information about sQTL.

Different types of molecular QTL were studied in the human brain. Besides sQTL (Qi et al. 2022; Takata et al. 2017; Zhang et al. 2020) and eQTL (O’Brien et al. 2018), methylation QTL (mQTL) (Gibbs et al. 2010; Ng et al. 2017) were also investigated to integrate epigenetic biology with gene expressions. Some of these molecular QTL were found to target biomarkers for neuropsychiatric disorders, and these efforts essentially have constructed a roadmap for genetically regulated molecular mechanisms in the human brain. We also focused on the brain tissues as the brain has the richest alternative splicing events and particular functions, allowing us to subsequently link to particular phenotypes strongly related to the brain functions. More could be done by assessing all available tissue samples from GTEx Consortium; nevertheless, it would require substantially more computational resources. We also expect the heterogeneity test method THISTLE to gain further power when incorporating the XAEM algorithm in future studies. As the most collected tissue, whole-blood RNA sequencing data are available in multiple human cohorts. We foresee a consortium-based investigation of irQTL in larger consortia and potentially provide a comprehensive assessment of irQTL associated with various human complex traits and diseases.

## Supplementary Information

Below is the link to the electronic supplementary material.

** Supplementary Fig. 1 **Quantile-quantile plot of the *p*-value distribution of 100,000 randomly selected variants from the irQTL analysis. 100,000 *p*-value under the null were drawn from a uniform distribution between 0 and 1. The median of the distribution is marked and labeled with the estimated inflation factor ${\uplambda }_{0.5}$ (PDF 2389 KB)

**Supplementary Fig. 2 Concordance between the annotated irQTL and established functional annotations.** The variants with v1.2 baseline annotations in the LDSC software were analyzed. The heatmap shows the 95% confidence interval lower bound of the odds ratio between irQTL and the functional annotations, i.e., the odds of an irQTL compared to other variants of being within the functional annotation (PDF 19 KB)

**Supplementary Fig. 3 Number of potential causal isoform biomarkers from MR for the UKB disease phenotypes.** GWAS summary statistics for 200 disease phenotypes with ICD codes were used in the MR analysis. The results with FDR less than 0.05 were counted (PDF 6 KB)

**Supplementary Fig. 4 Uniform** ***p*-value distribution of the reverse causal inference analysis.** To check reverse causality, generalized summary-statistics-based MR (GSMR) was applied to the complex traits as exposures and the isoforms as outcomes, where the cis-irQTL-based MR estimates were statistically significant. Fifteen randomly selected isoforms (for about 10% of the MR discoveries) were analyzed (PDF 150 KB)

**Supplementary Fig. 5 Quantile-quantile plot for the heritability enrichment signals across all the investigated tissue-trait associations.** The significant enrichment tests with a false discovery rate less than 0.05 were highlighted in blue (PDF 969 KB)

**Supplementary Table 1 List of significant irQTL for the 13 brain regions. **chr: chromosome; pos: base pair position; ref allele: reference allele; effect allele: coding allele; maf: minor allele frequency; n: sample size; beta: effect size; se: standard error; *p*: *p*-value (XLSX 269 KB)

**Supplementary Table 2 The number of mapped irQTL in each tissue compared to that of established sQTL.** The irQTL lead variants were cross-referenced in the GTEx v8 sQTL analysis, and the irQTL that were also genome-wide significant sQTL were counted (XLSX 9 KB)

**Supplementary Table 3 Types of the genes involved in the discovery of irQTL**. This gives an overview of the distribution of gene biotype groups across the genes used in irQTL analysis. A complete list of the genes and their corresponding isoforms is also provided (XLSX 743 KB)

** Supplementary Table 4 Stratified LD score regression analysis for the enrichment of neuro-related complex traits’ heritabilities at the reported irQTL.** Enrichment_p and Enrichment_p_1s are the two-sided and one-sided *p*-values for the heritability enrichment fold parameter, and FDR the corresponding false discovery rate calculated based on all the one-sided *p*-values in the same tissue, testing whether the enrichment fold is greater than 1 (XLSX 273 KB)

**Supplementary Table 5 Summary of the significant MR results for neuro-related phenotypes.** Significance was determined as a false discovery rate of less than 5%. Effect: irQTL effect; SE: standard error for the irQTL effect; beta: genetic effect on the complex trait; se: standard error for beta; MR effect: causal effect estimate of the isoform on the trait based on MR; MR se: standard error for the MR effect (XLSX 351 KB)

**Supplementary Table 6 Summary of the significant MR results for UKB disease phenotypes.** Significance was determined as a false discovery rate of less than 5%. Effect: irQTL effect; SE: standard error for the irQTL effect; beta: genetic effect on the complex trait; se: standard error for beta; MR effect: causal effect estimate of the isoform on the trait based on MR; MR se: standard error for the MR effect (XLSX 447 KB)

** Supplementary Table 7 Comparison of heritability enrichment at irQTL genes and other coding genes for the traits with significant S-LDSC discoveries.** Results for irQTL genes annotations are labeled with “irQTL”. Results for the other coding genes annotations are labeled with “other”. The last three columns give the enrichment fold estimates at irQTL genes relative to the other coding genes, where the standard errors were computed using the Delta method.2 (XLSX 14 KB)

## Data Availability

Codes to reproduce the results are deposited in Github (https://github.com/eudoraleer/Code_for_Brain_Isoform).

## Abbreviations

cis-irQTL: Cis-isoform ratio quantitative trait loci
CRP: Cluster response profile
eQTL: Expression quantitative trait loci
FDR: False discovery rate
GENCODE: The GENCODE project for the identification and classification of all gene features in the human and mouse genomes with high accuracy
GTEx: Genotype-tissue expression
GWAS: Genome-wide association studies
ICD: International classification of diseases
IVW: Inverse-variance weighted
irQTL: Isoform-ratio quantitative trait loci
LD: Linkage disequilibrium
MAF: Minor allele frequency
MMAB: Metabolism Of Cobalamin Associated B
mQTL: Metabolites quantitative trait loci
MR: Mendelian randomization
PCs: Principal components
PCA: Principal component analysis
PLINK: PLINK is a whole genome association analysis toolset
PSI: Percent spliced index
pQTL: Protein quantitative trait loci
S-LDSC: Stratified linkage disequilibrium score regression
sQTL: Splicing quantitative trait loci
THISTLE: Testing for heterogeneity between isoform-eQTL effects
TPM: Transcripts per million
XAEM: X-matrix alternating expectation–maximization
UKB: UK Biobank

## Data Availability

RNA-Sequencing data and WGS data from GTEx Consortium were obtained from the dbGaP repository under accession phs000424.v7.p2.c1 and phs000424.v7.p1 respectively. GWAS summary statistics for complex traits and diseases were obtained from the LD-Hub (https://ldsc.broadinstitute.org/) and Neale’s lab UKB round2 GWAS summary-level data (http://www.nealelab.is/uk-biobank).
