# Supplementary figures and images for: Hidden Genetic Regulation of Human Complex Traits via Brain Isoforms
Source: Phenomics. 2023 Mar 20;3(3):217–27. doi: 10.1007/s43657-023-00100-6 (PMC10260721; doi:10.1007/s43657-023-00100-6)

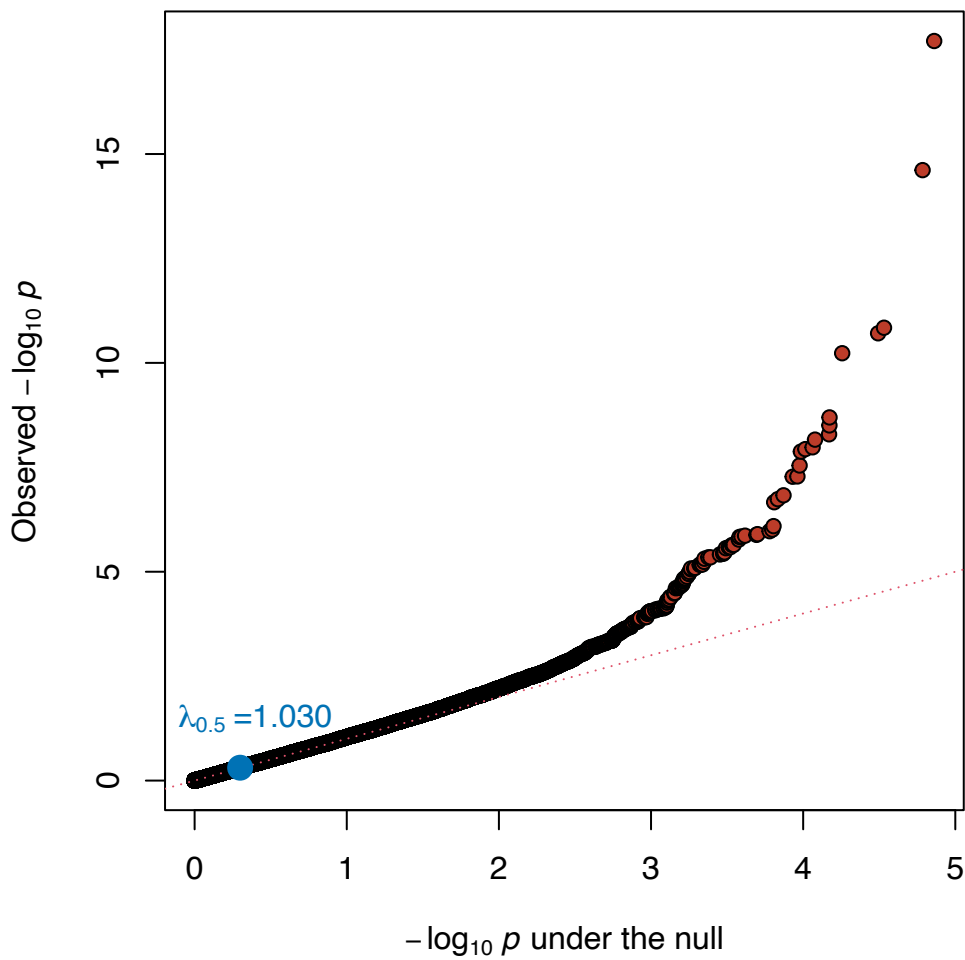

Supplement: Supplementary file 1 — Supplementary Fig. 1 Quantile-quantile plot of the p-value distribution of 100,000 randomly selected variants from the irQTL analysis. 100,000 p-value under the null were drawn from a uniform distribution between 0 and 1. The median of the distribution is marked and labeled with the estimated inflation factor ${\uplambda }_{0.5}$λ0.5 (PDF 2389 KB) [file 43657_2023_100_MOESM1_ESM.pdf]

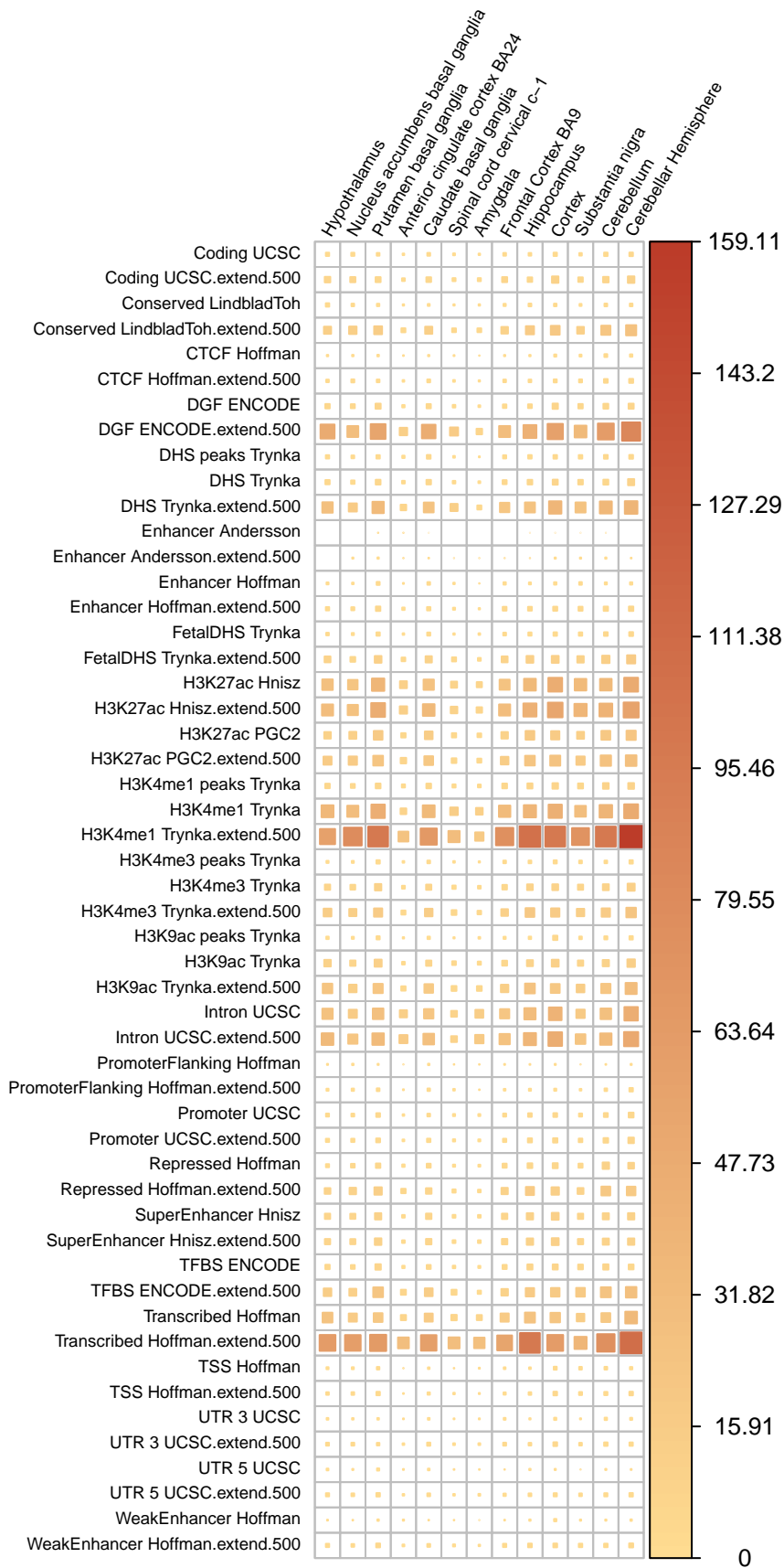

Supplement: Supplementary file 2 — Supplementary Fig. 2 Concordance between the annotated irQTL and established functional annotations. The variants with v1.2 baseline annotations in the LDSC software were analyzed. The heatmap shows the 95% confidence interval lower bound of the odds ratio between irQTL and the functional annotations, i.e., the odds of an irQTL compared to other variants of being within the functional annotation (PDF 19 KB) [file 43657_2023_100_MOESM2_ESM.pdf]

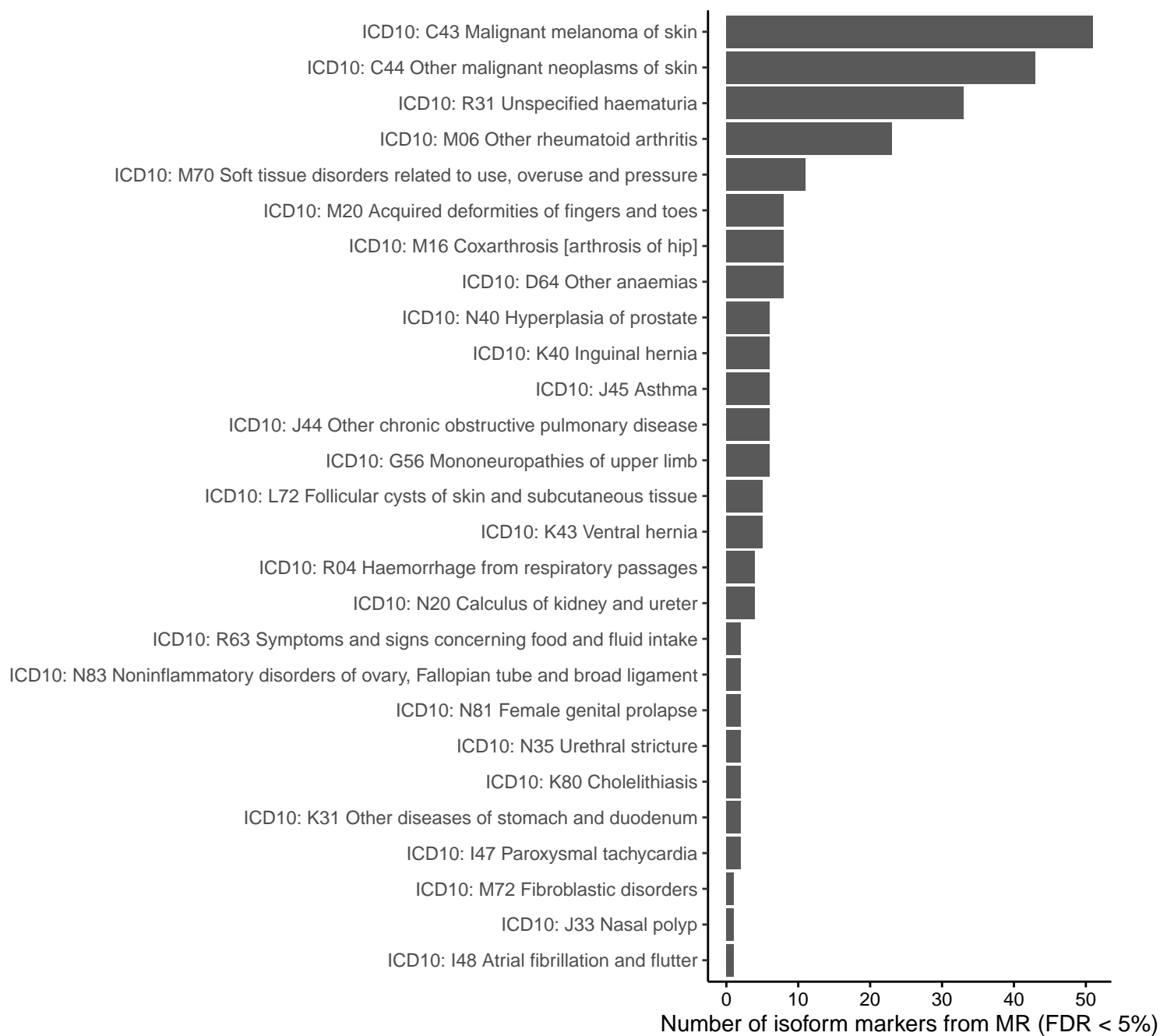

Supplement: Supplementary file 3 — Supplementary Fig. 3 Number of potential causal isoform biomarkers from MR for the UKB disease phenotypes. GWAS summary statistics for 200 disease phenotypes with ICD codes were used in the MR analysis. The results with FDR less than 0.05 were counted (PDF 6 KB) [file 43657_2023_100_MOESM3_ESM.pdf]

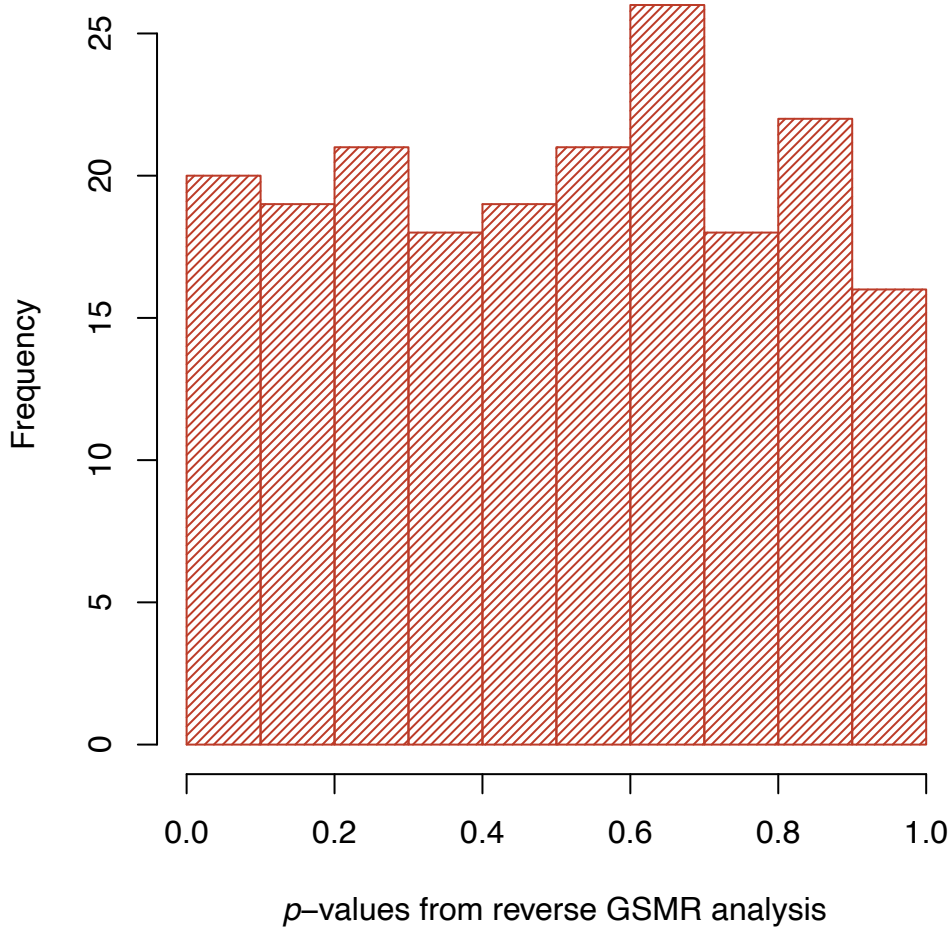

Supplement: Supplementary file 4 — Supplementary Fig. 4 Uniform p-value distribution of the reverse causal inference analysis. To check reverse causality, generalized summary-statistics-based MR (GSMR) was applied to the complex traits as exposures and the isoforms as outcomes, where the cis-irQTL-based MR estimates were statistically significant. Fifteen randomly selected isoforms (for about 10% of the MR discoveries) were analyzed (PDF 150 KB) [file 43657_2023_100_MOESM4_ESM.pdf]

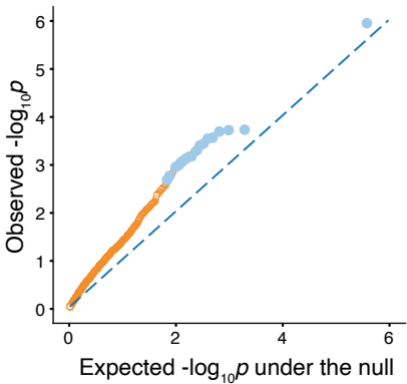

Supplement: Supplementary file 5 — Supplementary Fig. 5 Quantile-quantile plot for the heritability enrichment signals across all the investigated tissue-trait associations. The significant enrichment tests with a false discovery rate less than 0.05 were highlighted in blue (PDF 969 KB) [file 43657_2023_100_MOESM5_ESM.pdf]
